# Fungus *Aspergillus niger* Processes Exogenous Zinc Nanoparticles into a Biogenic Oxalate Mineral

**DOI:** 10.3390/jof6040210

**Published:** 2020-10-08

**Authors:** Martin Šebesta, Martin Urík, Marek Bujdoš, Marek Kolenčík, Ivo Vávra, Edmund Dobročka, Hyunjung Kim, Peter Matúš

**Affiliations:** 1 Institute of Laboratory Research on Geomaterials, Faculty of Natural Sciences, Comenius University in Bratislava, Mlynská dolina, Ilkovičova 6, 842 15 Bratislava, Slovakia; prif.ulg@uniba.sk (M.U.); marek.bujdos@uniba.sk (M.B.); peter.matus@uniba.sk (P.M.); 2 Department of Soil Science and Geology, Faculty of Agrobiology and Food Resources, Slovak University of Agriculture in Nitra, Trieda A. Hlinku 2, 949 76 Nitra, Slovakia; marek.kolencik@uniag.sk; 3 Nanotechnology Centre, VŠB Technical University of Ostrava, 17. listopadu 15/2172, 708 33 Ostrava, Czech Republic; 4 Institute of Electrical Engineering, Slovak Academy of Sciences, Dúbravská cesta 9, 841 04 Bratislava, Slovakia; ivo.vavra@savba.sk (I.V.); elekdobr@savba.sk (E.D.); 5 Department of Mineral Resources and Energy Engineering, Jeonbuk National University, 567, Baekje-daero, Deokjin-gu, Jeonju, Jeonbuk 54896, Korea; kshjkim@jbnu.ac.kr

**Keywords:** biotransformation, biomineralization, metal oxide nanoparticles, nanoparticle dissolution, nanoparticle mobility, fungal leaching

## Abstract

Zinc oxide nanoparticles (ZnO NPs) belong to the most widely used nanoparticles in both commercial products and industrial applications. Hence, they are frequently released into the environment. Soil fungi can affect the mobilization of zinc from ZnO NPs in soils, and thus they can heavily influence the mobility and bioavailability of zinc there. Therefore, ubiquitous soil fungus *Aspergillus niger* was selected as a test organism to evaluate the fungal interaction with ZnO NPs. As anticipated, the *A. niger* strain significantly affected the stability of particulate forms of ZnO due to the acidification of its environment. The influence of ZnO NPs on fungus was compared to the aqueous Zn cations and to bulk ZnO as well. Bulk ZnO had the least effect on fungal growth, while the response of *A. niger* to ZnO NPs was comparable with ionic zinc. Our results have shown that soil fungus can efficiently bioaccumulate Zn that was bioextracted from ZnO. Furthermore, it influences Zn bioavailability to plants by ZnO NPs transformation to stable biogenic minerals. Hence, a newly formed biogenic mineral phase of zinc oxalate was identified after the experiment with *A. niger* strain’s extracellular metabolites highlighting the fungal significance in zinc biogeochemistry.

## 1. Introduction

Engineered nanoparticles (ENPs) have been increasingly used in diverse applications concerning renewable energy, electronics, material science, medicine, and agriculture [1]. The increase in ENPs’ release to the environment also greatly raises the environmental risks in both frequency and severity [2]. This is especially true for soils and sediments, which represent the major sinks for released ENP. There, the mobility and transformation of ENP are strongly influenced by biogeochemical processes and, thus, they have been increasingly studied in the last two decades [3,4]. This also includes zinc oxide nanoparticles (ZnO NPs) of which, by estimate, up to 8.7 kt end up in soils annually [5].

Zinc oxide, in both bulk and nanosized form, has been extensively used in the industrial and commercial sphere with the estimated global annual production of 1337 and 30 kt in 2014, respectively [6]. Since then, the production of both bulk ZnO and ZnO NPs has shown a rising trend [7,8].

Two main pathways are important to ZnO NP entry to soil environments—(1) unintentional applications with activated sludges that are used as fertilizers, and (2) intentional direct application as a nanofertilizer to supply plants with Zn. Nanotechnology increases the growth and productivity of plants and is used to protect plants from pathogens, and is, thus, increasingly used in agriculture. Therefore, it is expected that the intentional application of ZnO NPs will play a larger role in soil contamination [9,10,11,12,13,14].

The mobility of ZnO NPs and other Zn forms in the soil is influenced by factors such as pH, the content and quality of clay minerals, oxyhydroxides of Al, Fe, Mn, and organic matter [15,16,17,18,19]. Living organisms also have a direct effect on bioavailability and the transformation of ZnO NPs. ZnO NPs have antimicrobial and antifungal properties and, hence, can affect the composition of microbial communities in soils and affect the growth of soil fungi [17,20,21,22].

Fungi may dissolve and transform ENPs, including ZnO NPs, and, under the right conditions, recreate them through biomineralization [23]. They have a big influence on the cycling of elements and the transformation of organic matter, with both processes heavily affecting the bioavailability of the elements in soils. Together with other soil organisms, they mechanically and chemically interact with soil particles and enhance weathering. This results in the translocation of trace elements from the solid phase to soil solutions. Thus, these elements become bioavailable to plants and other soil organisms.

Thanks to their ubiquitous activity, fungi have a considerable role in biogeochemical cycles of different elements, including Zn [24]. Fungal-mediated mineralization is ubiquitous in nature and has been used in industrial, biotechnological, and environmental applications [25]. Biomineralization also affects the ZnO NPs released to soil environments and has not been thoroughly studied.

Many fungi, including *Aspergillus niger*, were observed to produce extracellular metabolites capable of complexolysis and/or ligand-promoted dissolution [26,27]. The amino acids, carboxylic acids, phenolic compounds, and siderophores are some known metabolites with the aforementioned properties [28]. Among the strong chelating agents, oxalic acid was shown to be produced in large quantities by *A. niger* and other fungi [29,30]. The extraction efficiency of oxalic acid is pH-dependable and increases with decreasing pH [31]. Thus, it is possible that, in soil microenvironments close to the fungi, oxalic acids and protonation of the environment dissolve Zn, and the Zn is locally redistributed and reprecipitates as Zn oxalate elsewhere [32].

To find out how ZnO NPs interact with filamentous fungus *A. niger* and how they differ from bulk and ionic Zn forms, *A. niger* was grown statically for 7 days in growth media enriched by 6.5 mg Zn∙L^−1^ in form of ZnSO_4_, ZnO NPs or bulk ZnO. Changes in the pH of the growth media and dry biomass were recorded. The influence of extracellular metabolites of *A. niger* was observed in a separate experiment, where the metabolites were applied on ZnO NPs and after 5 days, a transformation into biominerals was recorded.

## 2. Materials and Methods

### 2.1. Preparation of ZnO Nanoparticle Suspensions and ZnSO_4_ Solution

For the experiment with fungal growth, three Zn forms were used, ionic Zn in the form of a solution of ZnSO_4_, suspension of ZnO NPs, and bulk ZnO. Bulk ZnO was acquired in the form of powder (p.a. quality, Chemapol, Prague, Czech Republic). ZnO NP dispersion used in the experiment was purchased from Sigma Aldrich, St. Louis, MO, USA (<100 nm particle size (TEM), ≤40 nm Avg. part. size (APS), 20 wt. % in H_2_O). ZnO NPs used in this work were also used in the article by Kolenčík et al. [16], and additional characterization of the nanoparticles can be found there. Right before the experiment, the suspension of ZnO NPs with a concentration of 65 mg∙L^−1^ (1 mmol Zn∙L^−1^) was prepared by adding 65 µL of ZnO NP dispersion to a 200 mL volumetric flask that was filled to mark with distilled water. The suspension was then sonicated for 15 min in an ultrasonic bath. An ionic zinc solution of 65 mg∙L^−1^ (1 mmol Zn∙L^−1^) ZnSO_4_ was prepared by dissolving 0.2876 g ZnSO_4_∙7H_2_O (p.a. quality, CentralChem, Bratislava, Slovakia) in 1 L of distilled water.

### 2.2. Cultivation of Aspergillus niger

Microscopic filamentous fungus *Aspergillus niger* (Tiegh.), strain CBS 140837, originally isolated from the mercury-contaminated soil [33], was grown in Sabouraud growth medium (HiMedia, Mumbai, India) in 250 mL Erlenmeyer flasks using a 7-day static cultivation in a growth chamber (dark, 25 °C). Four different types of growth media were created with three forms of Zn at 6.5 mg Zn∙L^−1^ (0.1 mmol Zn∙L^−1^), ZnSO_4_, ZnO NP, and bulk ZnO, and one control without added Zn. The concentration of 6.5 mg Zn∙L^−1^ was selected in a preliminary experiment with ZnSO_4_ (Supplementary Material Table S1, Figure S1), where a concentration of 13 mg Zn∙L^−1^ in the form of ZnSO_4_ prolonged sporulation with negligible effect on dry biomass weight; a concentration of 26 mg Zn∙L^−1^ was inhibitory for fungal growth and no compact mycelium was formed after 7 days.

In the case of ZnSO_4,_ and ZnO NPs, 5 mL of either 1 mmol Zn∙L^−1^ ZnSO_4_ solution or ZnO NP suspension were added to 45 mL of Sabouraud growth medium in Erlenmeyer flask. Bulk ZnO in the form of 0.0033 g of ZnO powder was added to 45 mL of growth medium and 5 mL of sterilized distilled water put into Erlenmeyer flask. The control experiment was done in Erlenmeyer flasks filled with 45 mL of the growth medium and 5 mL of sterilized distilled water. Each of the Zn forms and control had six replicates. All the Erlenmeyer flasks with the growth media were then put into the ultrasonic bath for 15 min.

After the aforementioned procedure, each of the growth media in Erlenmeyer flasks was inoculated with 50 μL of *A. niger* spore suspension and grown in the dark in the growth chamber for 7 days. After a 7-day growth period, the weight of dry biomass, the concentration of Zn in dry biomass, pH in the growth media, and the concentration of Zn in growth media in form of ionic Zn and Zn bound in organic or inorganic colloids was measured.

The biomass grown on the top of the growth media was collected and washed several times with distilled water. Afterwards, it was dried out at 60 °C, then weighed, and transferred into PTFE containers and 5 mL of 65% HNO_3_ was added to digest the biomass. The PTFE containers were put into high-pressure acid digestion vessels, and the vessels were closed afterwards. Then, the vessels were placed into an oven heated to 150 °C for 4 h to digest the biomass.

To discern between Zn bound to colloidal form and ionic Zn, the removed growth medium was centrifuged at 700 g for 1 min, to remove big clusters of residual biomasses bigger than 1000 nm. Then, part of the supernatant was removed and analyzed for Zn concentration (*C*_1000_). The concentration of ionic Zn (< 1 nm, *C*_1_) was acquired after the ultrafiltration of the supernatant, 6 mL of supernatant was transferred to ultrafiltration centrifugation units (Sartorius Vivaspin^®^ 6 mL, 3 kDa, Goettingen, Germany) which were centrifuged at 3500 g for 20 min. 0.5 mL of filtrate was collected, stabilized with HNO_3_, and analyzed for Zn concentration. The concentration in filtrates, supernatants, and digested biomass was measured by flame atomic absorption spectrometry (Perkin-Elmer 1100, Perkin-Elmer, Rodgau, Germany). A concentration of Zn bound to colloidal forms (1–1000 nm, *C*_1-1000_) was calculated by subtracting the concentration of ionic Zn from the concentration of Zn in colloidal supernatant (*C*_1-1000_ = *C*_1000_ − *C*_1_).

The dry weight of mycelia and pH was compared for all applications via a two-tail *t*-test at a significance level α = 0.05. Before the *t*-test, data were analyzed for differences in variances by F-test, and then a *t*-test for either equal or unequal variances was used. The statistical evaluation was done with Analysis ToolPak add-in for Microsoft Excel (Redmond, WA, USA).

### 2.3. Transformation of ZnO Nanoparticles by Extracellular Metabolites of Aspergillus niger

In our experiments with *A. niger* mycelia, the used concentration was very low, and most, if not all, the ZnO NPs and bulk ZnO were dissolved. Therefore, the experiment with fungal extracellular metabolites, which were also used in other studies of ZnO NPs synthesis [22], was undertaken to find out if these metabolites are able to dissolve ZnO NPs and transform them into Zn biominerals.

To achieve this, the *A. niger* was statically grown for 7 days on Sabouraud growth media in the growth chamber. It was grown the same way as the control group was grown in the previous experiment.

After the 7-day cultivation period, the mycelium was removed from the Erlenmeyer flask and was put for 3 days into 50 mL of sterilized distilled water. After 3-day cultivation, the mycelium was removed, and the acquired solution was filtered through 0.45 μm membrane filter paper. A small amount of the solution was removed and used for the analysis of oxalic acid produced by the fungal mycelium.

An Erlenmeyer flask was filled with 47.5 mL of a solution of extracellular fungal metabolites, and 500 mg of ZnO NPs was added in the form of 2.5 mL of ZnO NP dispersion. After 5 days of static reaction, the solution was decanted and analyzed for the content of oxalic acid and the white sediment was dried out and sent to X-ray powder diffraction (XRD) analysis and analysis by transmission electron microscopy (TEM). The solutions containing extracellular metabolites were analyzed for the content of oxalic acid by isotachophoresis (ZKI-1, Villa Labeco, Spišská Nová Ves, Slovakia) in itp-itp mode. The acquired isotachopherograms were evaluated by a software suite supplied with the analyzer [34].

### 2.4. Characterization of ZnO Nanoparticles and Bulk ZnO

Crystalline phases in ZnO NPs, bulk ZnO, and ZnO NPs transformed by the extracellular metabolites of *A. niger* were identified by XRD [35,36].

Surface morphology and the size of ZnO NPs, and bulk ZnO was examined by TEM. TEM images were collected on instrument JEOL-1200 EX (JEOL Ltd., Tokyo, Japan) operating at accelerating voltages of 120 kV. Samples of ZnO NPs and bulk ZnO were diluted in distilled water and ultrasonicated in order to break up large aggregates. A drop of the suspension was placed onto a carbon-coated grid and then air-dried at room temperature overnight. The ZnO NPs and bulk ZnO used here were also used and characterized in our previous study [9].

## 3. Results

### 3.1. Interactions of Aspergillus niger with ZnO and Aqueous Zn

**Figure 1:**
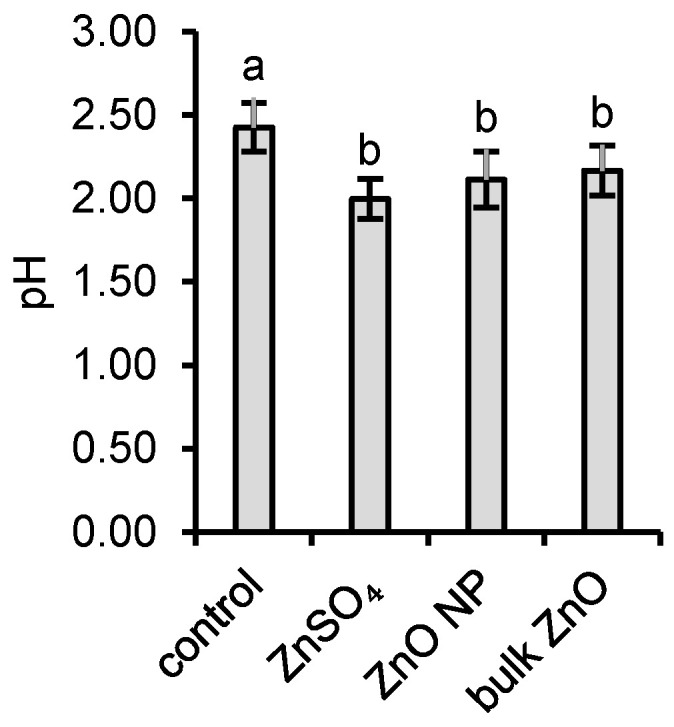
pH of the growth media after the experiment; a and b represent the statistically similar means between different groups at α = 0.05 (two-tail *t*-test).

Fungal mycelia of *Aspergillus niger* were grown statically in Erlenmeyer flasks with Sabouraud growth media spiked with either ZnSO_4_, ZnO NPs, or bulk ZnO and control without added Zn. The overproduction of protons (H^+^) by microscopic filamentous fungus *A. niger* during the 7-day cultivation period generated a substantial decrease of pH from 5.6 to 2.4. It was even more intensified in the presence of Zn where the final pH values decreased to 2.0, 2.1, and 2.2 for ZnSO_4_, ZnO NPs, and bulk ZnO, respectively (Figure 1).

**Figure 2:**
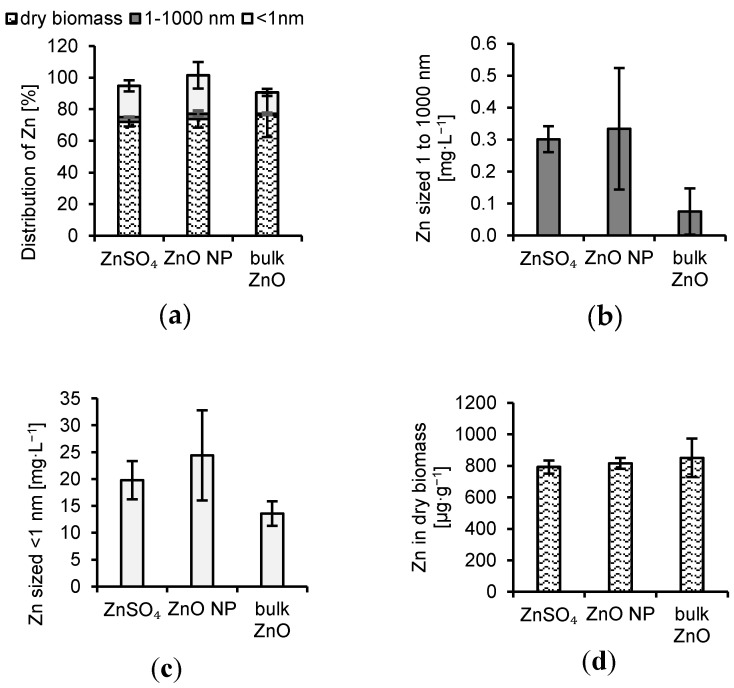
Distribution of Zn in the experimental system (**a**) and concentration of Zn in (**b**) particles sized 1 to 1000 nm, (**c**) dissolved fraction, and (**d**) dry biomass.

Zn was determined primarily as dissolved in the growth media (Figure 2) regardless of the Zn form applied. Only a negligible fraction was bound to colloidal particles. Thus, the distribution of Zn in colloidal, dissolved and bioaccumulated fractions were similar for ZnSO_4_, ZnO NPs, and bulk ZnO (Figure 2). The bioaccumulation of Zn by *A. niger* was very effective for all Zn forms (Figure 2) and represented up to 76% of the total Zn added to the cultivation system.

**Figure 3:**
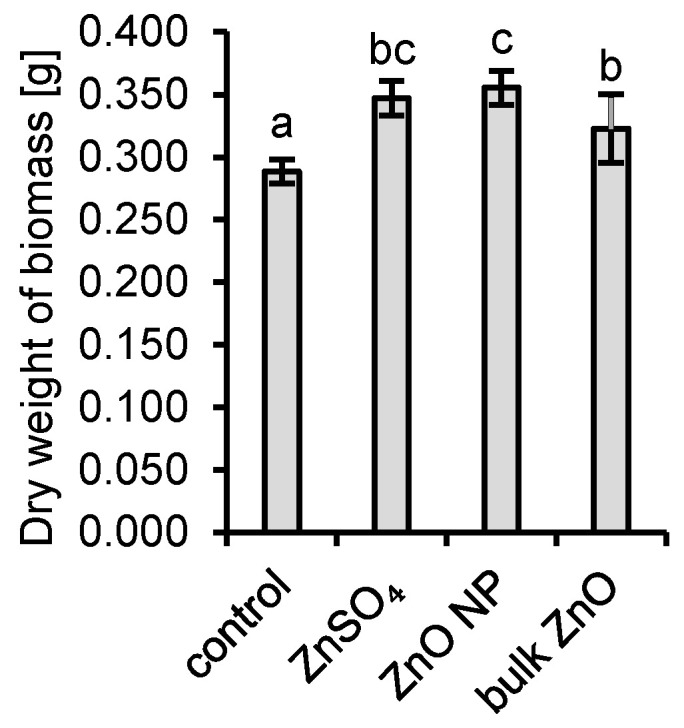
The dry weight of biomass of *Aspergillus niger* after the experiment; a, b and c represent the statistically similar means between different groups at α = 0.05 (two-tail *t*-test).

The applied concentration of 6.5 mg Zn∙L^−1^ had no adverse effects on fungal growth and a positive effect of Zn on the growth of the fungal biomass was observed (Figure 3). The application of all Zn form resulted in a higher dry weight of mycelia compared to control. The highest mean dry weight of mycelia was observed for ZnO NP application. The weight was significantly higher than both dry weights in bulk ZnO application and in the control experiment without additional Zn.

### 3.2. Transformation of ZnO Nanoparticles by Fungal Extracellular Metabolites

**Figure 4:**
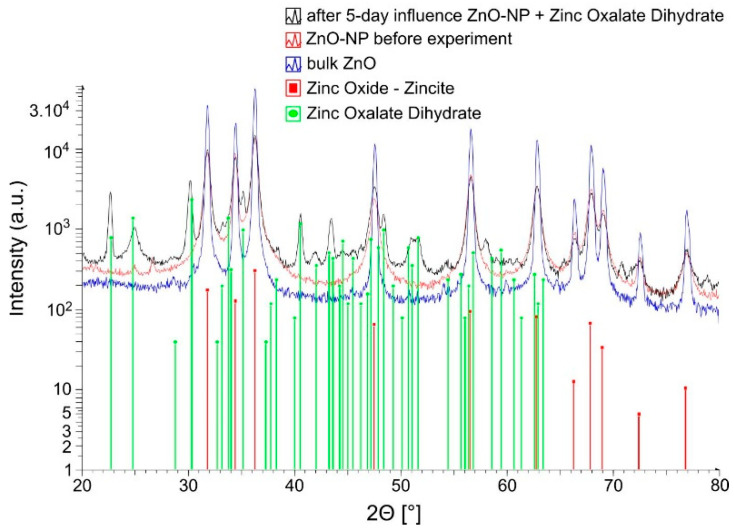
Diffractogram of the partially transformed ZnO NP to Zn oxalate dihydrate.

To experimentally confirm the transformation of ZnO NPs into zinc oxalate, extracellular metabolites of *A. niger* were applied to ZnO NPs. A partial transformation of ZnO NPs was observed, and a new mineral phase—zinc oxalate dihydrate—was identified (Figure 4).

**Figure 5:**
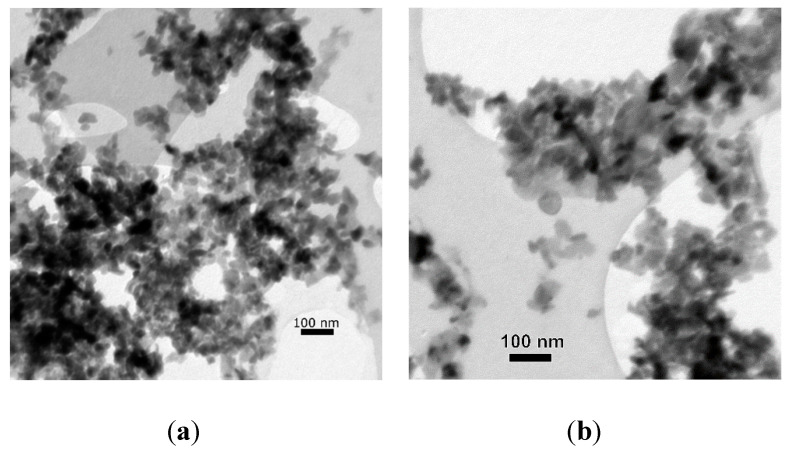
Transmission electron microscopy image of (**a**) Pristine ZnO nanoparticles (**b**) Partially transformed ZnO nanoparticles to Zn oxalate dihydrate.

At the end of the experiment, the oxalic concentration decreased from the initial 11.6 mmol∙L^−1^ to 1.2 mmol∙L^−1^. Thus, a 90% decrease in the initial oxalic acid concentration was observed, further confirming the ZnO NPs transformation into the zinc oxalate dihydrate by *A. niger*. Quantitatively, approximately 43 mg or 8.6% of ZnO NPs was transformed into the zinc oxalate dihydrate in the process. Only a partial transformation was observed. The recrystallization did not result in a significant change in the size of the most particles, and it ranged between 40 and 100 nm (Figure 5).

## 4. Discussion

### 4.1. Interactions of Aspergillus niger with ZnO and Aqueous Zn

ZnO NPs are relatively easily dissolvable in soils, especially in more acidic ones [9,37,38]. The dissolution of ZnO NPs is also enhanced by soil organisms, including filamentous fungi, via exudation of various acidic and chelating metabolites. The microbially induced acidification led to the dissolution of ZnO NPs, and bulk ZnO [39] and, therefore, nearly all of the Zn in the solutions of growth media was dissolved regardless of the Zn form.

Since Zn is an essential micronutrient [40,41] and our selected concentration was below the threshold of growth inhibition, all forms of Zn at the applied concentration had a positive effect on the dry weight of biomass after 7 days of cultivation. It was reported that the Zn concentrations as low as 0.065 mg Zn∙L^−1^ enhanced the fungal growth [42]. The minimal inhibitory concentration of Zn for microscopic filamentous fungi were reported to be as high as 100 mg Zn∙L^−1^ in the literature [43,44].

### 4.2. Transformation of ZnO Nanoparticles by Fungal Extracellular Metabolites

Filamentous fungi are capable of releasing extracellular metabolites that chemically deteriorate natural mineral Zn phases in soils and sediments via processes of protonation and chelation. However, under specific conditions (e.g., high biogenic chelate concentrations, alkalic pH), the bioextracted Zn can be reprecipitated from the soil solution to form new mineral phases. It has been reported that fungi react to the presence of ZnO NPs by increasing the production of the extracellular metabolites in order to detoxify and immobilize excessive Zn, and thus the transformation of ZnO NPs occurs in the soil environment [39,41]. Immobilization of Zn dissolved by fungi is facilitated by precipitation with oxalates [39].

We used extracellular metabolites of *A. niger* to transform ZnO NPs into a zinc oxalate biomineral. *A. niger* is well known for the high rate of extracellular metabolite production. Oxalic acid is the most produced of organic acids by the fungus when grown on Sabouraud growth media [30].

In our experiment, at the given applied volume and concentration of oxalic acid applied, only a partial transformation of ZnO NPs to zinc oxalates was observed. No significant change in the size of particles was observed. It is most probable that the transformation occurred on the surface of the nanoparticles, creating a surface of Zn oxalate with the core still being ZnO, or the newly formed zinc oxalates did not have time to grow to larger sizes in five days. If a larger volume of the organic acids was applied, both larger amounts of ZnO NPs might have been transformed, and the size of the particles could have seen a bigger change.

A change in Zn minerals into Zn oxalates had been observed before, albeit the minerals were not nanoparticles [32,39,45]. When silicate and sulfide minerals containing Zn were transformed by fungal extracellular metabolites, the resulting crystals of zinc oxalates had various shapes and sizes, with most of the crystals measuring between 50 and 100 µm [39]. When ZnO microparticulate powder was transformed into zinc oxalates by *Aspergillus* species, the biominerals had a different habitus compared to the minerals formed abiotically. The size of these biominerals was above 50 µm.

The mineral size and shape are also dependent on the place of formation. Minerals of zinc oxalate associated with a fungus have a different shape compared to both biomineral and abiotically formed Zn oxalate. However, the biominerals formed farther away from the fungal mycelium had a shape that was more similar to the abiotically formed Zn oxalate [32]. Since our experiment was only done with extracellular metabolites, the smaller change in the size and shape upon partial transformation may also result from the same processes that led to the increase in similarities between the biomineral formed further away from the mycelium and the abiotically formed mineral.

*Aspergillus* spp. and other fungi can biomineralize inorganic nanoparticles [46] and transform these nanoparticles into more stable oxalate compounds [25,47]. They play a large role in the cycling of elements in the soil environment, and their abilities can also be used for the bioremediation of contaminated areas [48].

## 5. Conclusions

Fungi, through their ability to acidify their environment, producing strong chelating agents, and the ability to bioaccumulate trace elements in their mycelia, can considerably affect the mobility of elements in soils and locally increase their bioavailability. Our observations affirm that the transformation of ZnO NPs into biogenic mineral phases occurs under the influence of ubiquitous soil fungus *A. niger* and that these processes may happen, under the right circumstances, in the soil environment. The role of soil fungi is seminal in the distribution of ionic and nanoparticulate forms of Zn. Therefore, fungi should be taken into consideration when models of bioavailability and mobility are constructed for soil environments. Furthermore, these fungal abilities can help us develop new methods of bioremediation in the future.

## Supplementary Materials

The following are available online at https://www.mdpi.com/2309-608X/6/4/210/s1, Figure S1: Photographs of Aspergillus niger mycelia after 7-day cultivation in the preliminary test, Table S1: Weight of dry biomass after 7-day static cultivation in the preliminary test.
