# Supplementary material for: Fungus Aspergillus niger Processes Exogenous Zinc Nanoparticles into a Biogenic Oxalate Mineral
Source: J Fungi (Basel). 2020 Oct 8;6(4):210. doi: 10.3390/jof6040210 (PMC7712133; doi:10.3390/jof6040210)
Supplement: Supplementary file 1 [file jof-06-00210-s001.pdf]

Supplementary Material

Manuscript title: Filamentous fungus *Aspergillus niger* transforms zinc oxide nanoparticles into oxalate biomineral

Authors: Martin Šebesta [a], Martin Urík [a], Marek Bujdoš [a], Marek Kolenčík [b], Ivo Vávra [c], Hyunjung Kim [d], Peter Matúš [a]

Affiliation:

[a] Institute of Laboratory Research on Geomaterials, Faculty of Natural Sciences, Comenius University in Bratislava, Mlynská dolina, Ilkovičova 6, 842 15 Bratislava, Slovak Republic

[b] Department of Soil Science and Geology, Faculty of Agrobiology and Food Resources, Slovak University of Agriculture in Nitra, Trieda A. Hlinku 2, 949 76 Nitra, Slovakia

[c] Institute of Electrical Engineering, Slovak Academy of Sciences, Dúbravská cesta 9, 841 04 Bratislava, Slovakia

[d] Department of Mineral Resources and Energy Engineering, Jeonbuk National University, 567, Baekje-daero, Deokjin-gu, Jeonju, 54896 Jeonbuk, Republic of Korea

Number of pages: 6

1. Preliminary *Aspergillus niger* growth experiment

1.1. Preparation of $ZnSO_4$ solutions

Stock ionic zinc solution of 1961 mg·l^{-1} (30 mmol Zn·l^{-1}) $ZnSO_4$ was prepared by dissolving 8.628 g $ZnSO_4 \cdot 7H_2O$ (p.a. quality, CentralChem, Bratislava, Slovakia) in 1 l of distilled water. Ionic zinc solutions of 654 mg·l^{-1} (10 mmol Zn·l^{-1}), 262 mg·l^{-1} (4 mmol Zn·l^{-1}), 131 mg·l^{-1} (2 mmol Zn·l^{-1}), 65 mg·l^{-1} (1 mmol Zn·l^{-1}), 33 mg·l^{-1} (0.5 mmol Zn·l^{-1}), and 6.5 mg·l^{-1} (0.1 mmol Zn·l^{-1}) were created by adding the appropriate volume of stock ionic zinc solution to 50 ml volumetric flask that was filled up to the mark with sterilized distilled water.

1.2 Preliminary cultivation of Aspergillus niger

Microscopic filamentous fungus *Aspergillus niger* (Tiegh.) was grown in Sabouraud growth medium (HiMedia, MH, India) in Erlenmeyer banks using 7-day static cultivation in a growth chamber (dark, 25°C). The growth media were spiked with several different concentrations of Zn, 196, 65, 26, 13, 6.5, 3.3, and 0.65 mg·l^{-1} (3, 1, 0.4, 0.2, 0.1, 0.05, and 0.01 mmol Zn·l^{-1}) by adding 5 ml of Zn solutions at the concentration of 1961, 654, 262, 131, 65, 33, and 6.5 mg·l^{-1} to 45 ml of Sabouraud growth medium in Erlenmeyer bank, respectively.

The control experiment was done in Erlenmeyer banks filled with 45 ml of the growth medium and 5 ml of sterilized distilled water.

Each of the Zn concentrations and control had 2 replicates. All the Erlenmeyer banks with the growth media were then put into the ultrasonic bath for 15 min.

Later, each of the growth media in Erlenmeyer banks was inoculated with spores of *A. niger* and grown in dark in the growth chamber for 7 days. After a 7-day growth period, the weight of dry biomass, the concentration of Zn in dry biomass, pH in the growth media, and the concentration of Zn in growth media in form of ionic Zn and Zn bound in organic or inorganic colloids was measured.

The biomass grown on the top of the growth media was collected and washed several times with distilled water. Afterward, it was dried out at 60°C, then weighed, and digested in an autoclave at 200°C by the mixture of concentrated acids and oxidizer (HNO_3, HCl, and H_2O_2). To discern between Zn bound to colloidal form and ionic Zn, the removed growth medium was centrifuged at 700 g for 1 min, to remove big clusters of residual biomasses bigger than 1000 nm. Then, part of the supernatant was removed and analyzed for Zn concentration. The concentration of ionic Zn was acquired after the ultrafiltration of the supernatant, 6 ml of supernatant was transferred to ultrafiltration centrifugation units (Sartorius Vivaspin® 6 ml, 3 kDa) which were centrifuged at 3500 g for 20 min. 0.5 ml of filtrate was collected, stabilized with HNO_3, and analyzed for Zn concentration. The concentration in filtrates, supernatants, and in digested biomass was measured by flame atomic absorption spectrometry (Perkin-Elmer 1100, Perkin-Elmer, Germany).

1.3 Results of preliminary experiment with Aspergillus niger and different concentrations of ionic Zn

Static cultivation of A. niger grown on Sabouraud growth media spiked with different concentrations of ionic Zn in the form of $ZnSO_4$ resulted in (nearly) total inhibition of growth above 13 $mg\cdot l^{-1}$ of Zn concentration (Table. S1). At 13 $mg\cdot l^{-1}$ mycelia retained similar although slightly lower dry weight compared to control. However, the sporulation was hindered at 13 $mg\cdot l^{-1}$ (Figure S1). A higher minimal inhibitory concentration of Zn at 100 mg $Zn\cdot L^{-1}$ for microscopic filamentous fungi was noted elsewhere (Sawai and Yoshikawa, 2004; Sardella et al., 2018).

References

Sardella, D., Gatt, R., Valdramidis, V.P., 2018. Assessing the efficacy of zinc oxide nanoparticles against Penicillium expansum by automated turbidimetric analysis. Mycology 9, 43–48. https://doi.org/10.1080/21501203.2017.1369187

Sawai, J., Yoshikawa, T., 2004. Quantitative evaluation of antifungal activity of metallic oxide powders (MgO, CaO and ZnO) by an indirect conductimetric assay. J. Appl. Microbiol. 96, 803–809. https://doi.org/10.1111/j.1365-2672.2004.02234.x

Table S1 Weight of dry biomass after 7-day static cultivation in the preliminary test

$ZnSO_4$ concentration [mmol·l^{-1}]	Zn concentration [mg·l^{-1}]	Weight of dry biomass ± standard deviation [g]
0.00	0.00	0.210 ± 0.014
0.01	0.65	0.255 ± 0.010
0.05	3.27	0.247 ± 0.008
0.10	6.54	0.257 ± 0.002
0.20	13.08	0.187 ± 0.032
0.40	26.15	n.a.
1.00	65.38	n.a.
3.00	196.14	n.a.

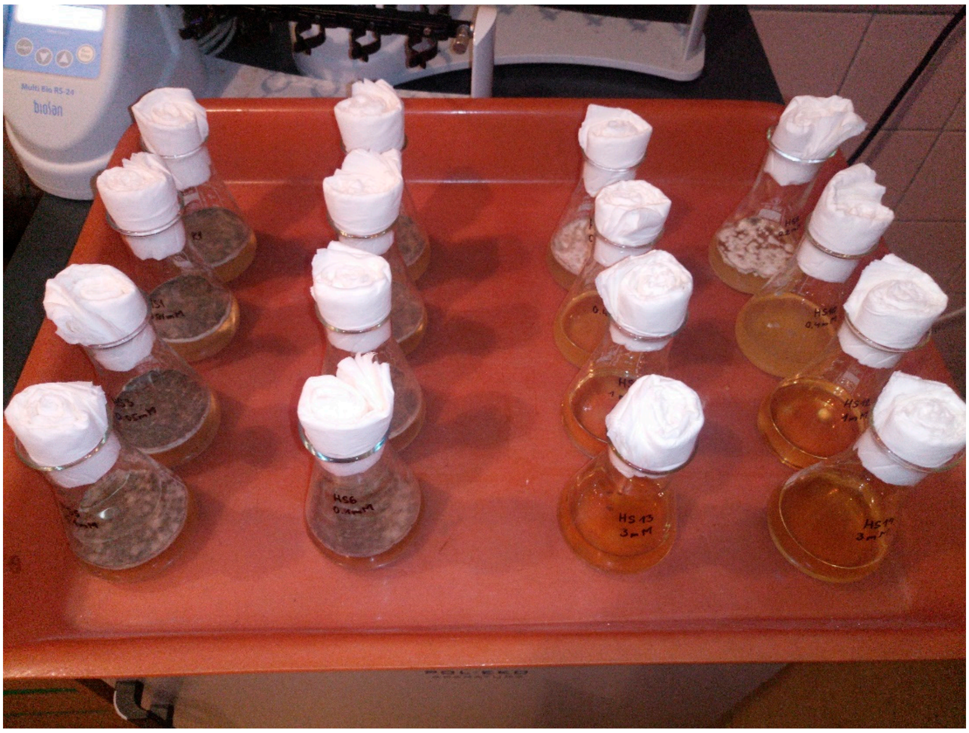

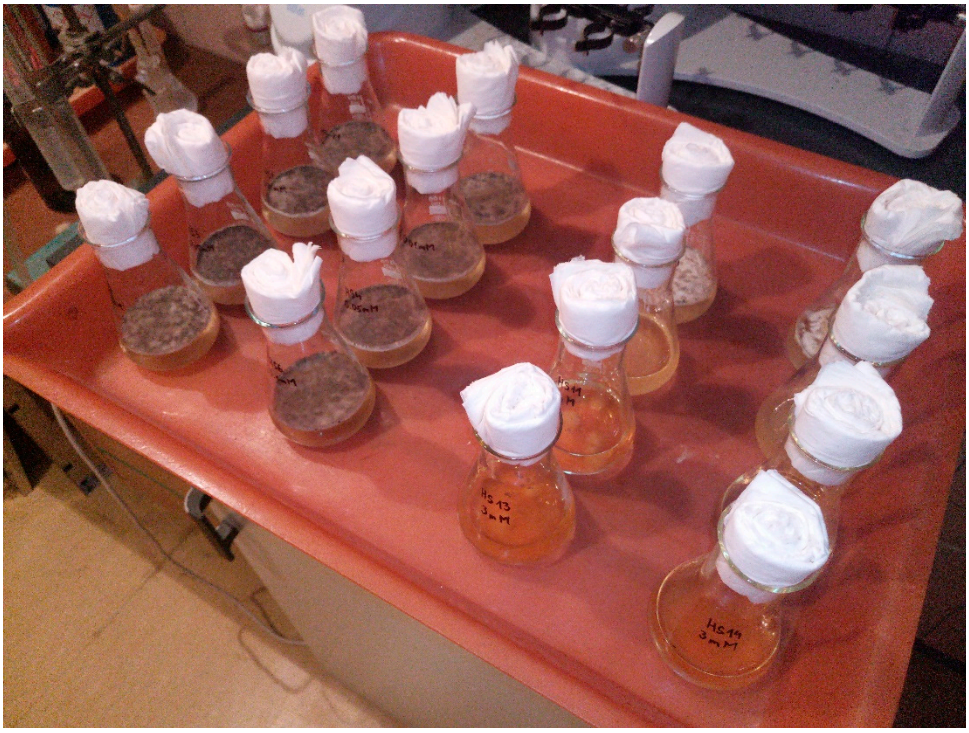

Figure S1 Photographs of *Aspergillus niger* mycelia after 7-day cultivation in the preliminary test
